# Mediating effect of sleep quality on the association between job stress and health-related productivity loss among workers in R&D enterprises in Shanghai

**DOI:** 10.3389/fpubh.2024.1331458

**Published:** 2024-01-24

**Authors:** Yixuan Sun, Minqi Wei, Qiuwen Zhao, Jinfeng Yang, Junling Gao, Junming Dai

**Affiliations:** ^1^School of Public Health, Fudan University, Shanghai, China; ^2^Administration Office, Minhang District Center for Disease Control and Prevention, Shanghai, China

**Keywords:** job stress, sleep quality, health-related productivity loss, path analysis, enterprise workers

## Abstract

**Background:**

Past research indicates that occupational stress negatively predicts health-related productivity. Simultaneously, sleep problem among workers may stem from job stress, subsequently leading to a decline in sleep quality and resulting in reduced health productivity. Therefore, this study aims to idenitify whether the sleep quality of employees functions as a mediator in the process through which job stress impacts health productivity.

**Objectives:**

This study aimed to assess the status and analyze differences in quality of sleep, job stress, and health-related productivity loss (HRPL) among workers in research and development (R&D) enterprises in Minhang District, Shanghai. We also assessed the mediating effect of sleep quality on the relationship between job stress and HRPL.

**Methods:**

A total of 3,216 workers in R&D firms aged between 18 and 60 years participated in this study (mean age 35.15 years; standard deviation 8.44; male-to-female ratio≈2:1). The Nakata Insomnia Questionnaire, the Chinese version of the Brief Job Stress Questionnaire revised edition, and the Chinese version of the Work Productivity and Activity Impairment Questionnaire were used in this study. And the Kruskal–Wallis test, Hierarchical Multiple Regression Analysis, and Path Analysis were utilized for data analysis in this study.

**Results:**

There were significant differences in the positive detection rate of insomnia among participants according to age, educational level, marital status, position, length of service, and level of financial difficulties (all *P* < 0.05). We also found significant differences in the positive detection rate of HRPL among participants according to age, marital status, length of service, and level of financial difficulties (all *P* < 0.05); participants with insomnia scored higher for HRPL than those without insomnia (6.00 vs. 4.20, *P* < 0.001). Additionally, participants with job stress problems had higher HRPL than those without these issues (7.00 vs. 4.20, *P* < 0.001). Our findings suggest that sleep quality plays a mediating role between job stress and HRPL (all *P* < 0.05).

**Conclusions:**

Occupational health professionals must pay particular attention to job stress, sleep quality, and their influencing factors to positively influence the wellbeing of workers while improving productivity.

## 1 Introduction

Job stress and insomnia, which are common health problems among enterprise workers, can lead to absenteeism and presenteeism, leading to health-related productivity loss (HRPL) (1).

Research indicates that the impact of job stress and sleep disorders on health productivity loss is multifaceted rather than singular (2). Job stress is demonstrated to adversely affect HRP (3), whereas sleep disorders also exhibit a detrimental influence on HRP, often triggered by job stress (4).

Understanding the antecedents of job stress, insomnia, and HRP among enterprise workers and the relationship between them is an important research topic. In this study, our aim was to explore the occupational factors associated with mental health and assess whether sleep quality as a mediator between job stress and HRPL. This analysis sought to examine the potential impact of job stress and sleep quality on HRP, enhancing the understanding of their predictive factors and health implications.

### 1.1 Job stress

In evaluating job stress models, the Job Demand-Control (JDC) model has been widely utilized among employees (5). In previous research, the JDC model has been employed to forecast insomnia (6). Moreover, Dai Junming conducted a study on the applicability of the JDC model among the workforce in Shanghai, China, developing a well-suited Chinese-translated questionnaire tailored to the cultural context (7). Therefore, this study will employ the JDC model to assess the level of job stress among the study subjects.

According to the job demand-control model (JDC), job stress is an adverse physiological and psychological response to an imbalance between job demands and control (include job decision latitude and skill), as well as an unsatisfactory level of individual controls (8).

Continued exposure to high-level job stress can produce a negative impact on workers' psychological and physical health (9). For example, several studies have shown that high job stress is a risk factor for insomnia (5), and others have shown that low job control is a more sensitive predictor of sleep disturbance (10), which occurs particularly often in industries such as chip and electronic manufacturing. Job stress can also cause damage to HRP (11) and is detrimental to personal wellbeing (12).

### 1.2 Quality of sleep

Insomnia and sleep deprivation are major health problems that plague the global population. Some studies (13) have shown that the sleep quality of workers, especially those performing mental tasks, is poorer than the norm in China. Difficulty falling asleep, light sleep, waking easily, and insufficient sleep duration are all forms of sleep disturbance. The observation that job stress adversely impacts sleep quality has been reiterated in various literature. Drawing from prior investigations involving occupational groups such as assembly line workers (14), grid employees (15), and civil servants (16), it is evident that chronic job stress within these populations tends to provoke sleep disturbances.

Sleep deprivation can lead to physiological changes in the immune system, psychological disorders, and reduced quality of life and concentration and it can be quite detrimental to the health of occupational workers (4).

### 1.3 Health-related productivity loss (HRPL)

HRPL is defined as “health completely preventing a person from working” or “health limiting a person's work performance” (17). When an employee has a health problem, they may choose to take time off of work to seek medical care (work absence) or they may go to work while ill. Absenteeism and presenteeism are collectively known as HRPL, which is a health economics concept that refers to the economic loss due to an employee's health problem. For both employees and employers, there are urgent reasons to be concerned about HRPL. First, for workers, impaired health productivity may result in direct medical expenses as well as indirect losses in the form of absence from work or reduced productivity (18). For companies, this is even more direct in terms of increased production costs and economic losses (19).

**Figure 1:**
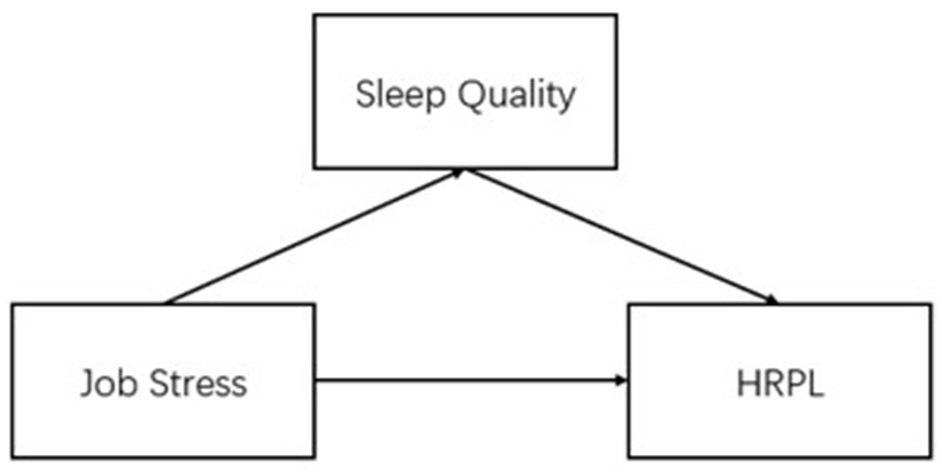
Conceptual model and main variables.

Previous studies have shown that psychosocial resources at work are the most important predictor of health-related productivity losses (3). Meanwhile, job stress is a predictor of insomnia and a variety of adverse health outcomes (20), and insomnia may contribute to decreased productivity and absenteeism (21). The simple relationship between any two of job stress, sleep quality and HRPL has been studied, but the way in which they work together is unclear. Therefore, we hypothesize whether sleep quality serves as a link in the pathway through which job stress influences HRP levels. To delve into this question, the hypothesis of this study is that sleep quality may play a mediating role in the relationship between occupational stress and HRPL. The theoretical model for this study is shown in Figure 1.

### 1.4 Objectives

Studies exploring the mediating effect of the association between occupational and HRPL remain scarce. Therefore, the purpose of this study was: (1) to assess the quality of sleep, job stress, and HRPL among workers in research and development (R&D) companies working in Minhang District, Shanghai; (2) to explore the predictive effect of sleep quality and job stress on HRPL; and (3) to assess the mediating effect of sleep quality on the relationship between job stress and HRPL.

## 2 Materials and methods

### 2.1 Measurement instruments

#### 2.1.1 Quality of sleep

The sleep quality measurement instrument in this study was based on Nakata's insomnia questionnaire (22). This tool includes three items: time to falling asleep (“How long do you usually fall asleep after going to bed?”), sleep depth(“Do you have situations that you tend to wake up easily after falling asleep?”), and morning awakening time in the previous month(“Have you ever woken up too early and found it difficult to fall asleep again?”). Insomnia is defined as the presence of one of three criteria: excessive time to falling asleep (≥30 min), insufficient sleep depth (more than three times per week), and early morning awakening (more than three times per week). All items were scored on a five-point Likert scale ranging from 5 to 1, with higher scores indicating better sleep quality. The Cronbach's α was 0.720, demonstrating good internal reliability.

#### 2.1.2 Job stress

The job content questionnaire was developed based on the JDC model by Karasek et al. The Chinese version of the Brief Occupational Stress Questionnaire was compiled by Dai et al. (7). The questionnaire consists of three dimensions with 16 items: job demands (e.g., “My job requires speed”), control over work (e.g., “I have the freedom to decide how to do my job”), and social support level (e.g., “My family is very supportive of my work”). All items were scored on a five-point Likert scale ranging from 1 (totally disagree) to 5 (totally agree). According to the JDC theory, the ratio of the mean number of job demands to control over work is used to define the level of occupational stress. If the ratio ≥1.0, the participant is defined as being in a state of occupational stress, and vice versa. The Cronbach's α was 0.813, demonstrating good internal reliability.

#### 2.1.3 Health-related productivity loss (HRPL)

A modified version of the Work Productivity and Activity Impairment Questionnaire (23) was used, consisting of three main questions.

① During the past 7 days, how many hours did you actually work? (no. hours).
② During the past days, how many hours did you miss from work because of your health problems? (no. hours).
③ During the past 7 days, how much did your health problems affect your productivity while you were working? (1–6, 8–11).

We calculated the number of work hours lost owing to health problems as an indicator of HRPL based on the answers to the three questions above, i.e., ①×(③/10)+②.

### 2.2 Procedures

In November–December 2021, seven enterprises with R&D departments were selected for this cross-sectional study using a convenience sampling method based on geographical distribution in conjunction with the overall development layout of Minhang District. The study was conducted using an online questionnaire with the assistance of each company. Participation was voluntary and the first page of the questionnaire explained the purpose of the study and informed participants how to complete the survey, how to withdraw from the study, and how to contact the investigators for more information. Respondents were also asked to read and sign an informed consent form.

A total of ~3,489 questionnaires were collected and 3,216 responses were ultimately included in the analysis (92.18% effective rate). The survey was administered in accordance with all ethical principles regarding informed consent, anonymity, and confidentiality. Inclusion criteria were as follows: (1) age between 18 and 60 years; (2) having worked in the enterprise for more than 6 months, (3) currently engaged in professional activities, and (4) signing the informed consent form. Ethical approval for this study was granted by the Ethics Committee of the Center for Disease Control and Prevention of Minhang District, Shanghai (code EC-P-2021-003).

### 2.3 Data analysis

Descriptive statistics were performed and data were reported using mean and standard deviation (SD) or frequency and percentage, with skewed distributions of HRPL, described using median and quartile. The normality of the data was assessed and confirmed using the Kolmogorov–Smirnov test. Due to the skewed distribution of HRPL, to assess whether differences existed between multiple groups, the Kruskal–Wallis test was used. To assess the association between variables, Pearson correlation coefficients were measured. Hierarchical multiple regression analysis was performed. Finally, this study employed path analysis to explore the causal chain mediated by sleep quality, aiming to elucidate the underlying mechanism between work stress and HRP levels. To avoid Type I errors, Bonferroni correction tests were conducted. To measure multicollinearity, we used a variance inflation factor (VIF = 1), which indicated that the variables were uncorrelated. All statistical procedures were performed using IBM SPSS 26 (IBM Corp., Armonk, NY, USA) and Mplus 8.3(MUTHEN & MUTHEN, 3463 Stoner Ave. Los Angeles, CA).

## 3 Results

A total of 3,216 active workers in R&D enterprises in Minhang District, Shanghai, aged between 18 and 60 years, participated in this study (median age 35.15 years; SD 8.44). Most participants were men (66.67%), married (71.33%), had a senior secondary degree (33.52%), and had persistent financial difficulties (54.32%). With regard to work-related characteristics, most participants were contract workers (88.71%); the ratio of manual workers (production and operations) to mental workers (R&D and administration) was approximately 1:1, and the average length of service was 12.18±9.25 years. Table 1 presents participants; sociodemographic characteristics in further detail.

**Table 1:** Sociodemographic characteristics, job stress, sleep quality, and health-related productivity loss of participants (*N* = 3,216; median age 35.15 years; SD 8.44).

		***n***	**%**	**Mean ±SD/median (25% quantile, 75% quantile)**
Sex	Female	2,144	66.67	-
	Male	1,072	33.33	-
Age, y	18–24	361	11.23	-
	25–34	1,360	42.29	-
	35–44	1,075	33.43	-
	45–60	420	13.06	-
Educational attainment	Junior secondary education and below	576	18.00	-
	Senior secondary degree/Technical secondary degree	1,078	33.52	-
	Junior college degree	499	15.52	-
	Undergraduate degree	808	25.12	-
	Postgraduate degree and above	255	7.93	-
Marital status	Married	2,294	71.33	-
	Unmarried (Single, Divorced/Widowed/other)	922	28.67	-
Household registration	Shanghai Household Registration	1,143	35.54	-
	Shanghai residence permit	873	27.15	-
	Neither	1,200	37.31	-
Years of service	<5	724	22.51	-
	5–10	899	27.95	-
	11–20	1,208	37.56	-
	>20	385	11.97	-
Employment status	Contract worker	2,853	88.71	-
	Labor dispatcher	150	4.66	-
	Other	213	6.62	-
Sector of activity	Administration	898	27.92	-
	Research and development	375	11.66	-
	Production and operations	1,513	47.05	-
	Other	430	13.37	-
Financial difficulties	Persistent	1,747	54.32	-
	Sometimes	1,103	34.30	-
	No difficulties	366	11.38	-
Job stress indicator	Job demands	-	-	3.21 ± 0.75
	Control over work	-	-	3.02 ± 0.70
	Job demands/decision latitude	-	-	1.12 ± 0.46
Job stress	Yes	1,780	55.35
	No	1,436	44.65
Sleep quality		-	-	9.81 ± 3.03
Insomnia	Yes	1,323	41.14
	No	1,891	58.80
HRPL	Absenteeism and Presenteeism (hours)	-	-	5.30 (0.00, 18.40)

Table 1 also shows the results of statistical analysis regarding job stress, sleep quality, and HRPL among workers in R&D companies. Job stress and sleep problems were evident among participants, with 41.14% having sleep problems and 55.35% having occupational stress. Participants' mean sleep quality score was 9.81 ± 3.03. The number of hours of HRPL was 5.30 (0.00, 18.40).

Table 2 shows the results for participants' levels of job stress, as well as sleep quality and levels of HRPL, by different demographic characteristics. There were significant differences in the positive detection rate of job stress among participants according to sex, educational level, marital status, position, length of service, and level of financial difficulties (all *P* < 0.05). Additionally, there were significant differences in the positive detection rate of insomnia among participants according to age, educational level, marital status, position, length of service, and level of financial difficulties (all *P* < 0.05). We found statistically significant differences in the positive detection rate of HRPL among participants for age, marital status, length of service, and level of financial difficulties (all *P* < 0.05). Table 2 also shows that participants with insomnia scored higher for HRPL than those without insomnia, and participants with job stress problems had higher HRPL.

**Table 2:** Comparison of sleep quality, job stress, and health-related productivity loss among workers in R&D companies with different characteristics.

	**n (%)**	**Job stress**	**Sleep quality**	**HRPL**
**Sex**				
Female	2,144 (66.67)	46.1	59.65	5.40 (0.00,20.00)
Male	1,072 (33.33)	41.8	57.20	5.20 (0.00,16.95)
X^2^		5.325	1.781	0.416
*P*		**0.021**	0.182	0.519
**Age, y**				
18–24	361 (11.23)	39.9	51.80	7.00 (0.00,24.00)
25–34	1,360 (42.29)	45.0	59.34	5.60 (0.00,19.15)
35–44	1,075 (33.43)	46.8	60.52	5.00 (0.00,18.00)
45–60	420 (13.06)	42.1	58.95	1.80 (0.00,14.40)
X^2^		6.440	8.781	22.645
*P*		0.092	**0.032**	**<0.001**
**Educational attainment**				
Junior secondary education and below	576 (18.00)	42.88	57.81	4.80 (0.00,18.00)
Senior secondary degree/Technical secondary degree	1,078 (33.52)	48.33	61.69	5.20 (0.00,20.00)
Junior college degree	499 (15.52)	44.09	63.05	4.20 (0.00,16.80)
Undergraduate degree	808 (25.12)	41.34	56.51	6.40 (0.00,18.40)
Postgraduate degree and above	255 (7.93)	44.71	48.24	5.30 (0.00,17.00)
X^2^		10.290	21.167	1.654
*P*		**0.036**	**<0.001**	0.437
**Marital status**				
Married	2,294 (71.33)	45.77	60.86	4.75 (0.00,16.80)
Unmarried (Single/Divorced/Widowed/other)	922 (28.67)	41.87	53.80	7.00 (0.00,22.00)
X^2^		4.060	13.561	16.824
*P*		**0.044**	**<0.001**	**<0.001**
**Household registration**				
Shanghai Household Registration	1,143 (35.54)	43.22	58.01	6.00 (0.00,18.00)
Shanghai residence permit	873 (27.15)	46.85	59.24	4.20 (0.00,16.90)
Neither	1,200 (37.31)	44.42	59.33	5.60 (0.00,20.00)
X^2^		2.682	0.508	5.401
*P*		0.262	0.776	0.067
**Years of service**				
<5	724 (22.51)	39.78	52.76	6.00 (0.00,21.60)
5–10	899 (27.95)	44.27	56.90	5.60 (0.00,18.00)
11–20	1,208 (37.56)	48.10	63.88	4.80 (0.00,18.00)
>20	385 (11.97)	43.90	58.96	4.00 (0.00,16.00)
X^2^		12.896	25.080	14.259
*P*		**0.005**	**<0.001**	**0.003**
**Employment status**				
Contract worker	2,853 (88.71)	44.48	58.56	5.40 (0.00,18.55)
Labor dispatcher	150 (4.66)	48.67	62.42	2.30 (0.00,15.25)
Other	213 (6.62)	44.13	60.09	6.30 (0.00,18.20)
X^2^		1.036	1.020	3.472
*P*		0.596	0.600	0.176
**Sector of activity**				
Administration	898 (27.92)	41.09	56.79	5.00 (0.00,16.80)
Research and development	375 (11.66)	40.53	49.87	8.00 (0.00,20.00)
Production and operations	1,513 (47.05)	49.44	61.90	5.20 (0.00,20.60)
Other	430 (13.37)	38.84	60.14	4.45 (0.00,16.00)
X^2^		27.088	20.184	5.610
*P*		**<0.001**	**<0.001**	0.132
**Financial difficulties**				
Persistent	1,747 (54.32)	50.60	65.25	6.00 (0.00,20.00)
Sometimes	1,103 (34.30)	38.98	52.50	4.70 (0.00,16.80)
No difficulties	366 (11.38)	33.33	47.27	4.00 (0.00,16.00)
X^2^		58.325	68.205	9.892
*P*		**<0.001**	**<0.001**	**0.007**
**Sleep disorders**				
Yes	1,891 (58.80)	39.53	-	4.20 (0.00,16.00)
No	1,323 (41.14)	48.23	-	6.00 (0.00,20.00)
X^2^		23.824	-	8.281
*P*		**<0.001**	-	**0.004**
**Job strain**				
Yes	1,436 (44.65)	-	55.03	4.00 (0.00,14.40)
No	1,780 (55.35)	-	63.55	7.20 (0.00,23.35)
X^2^		-	23.824	40.440
*P*		-	**<0.001**	**<0.001**

Bold values mean *p* < 0.05.

A correlation matrix was created using all variables to assess the associations among job stress, sleep quality, and HRPL. As displayed in Table 3, significant correlations were found for all variables.

**Table 3:** Correlation analysis between job stress, sleep quality, and health-related productivity loss among workers in R&D companies.

	**1**	**2**	**3**	**4**	**5**
1. HRPL	1				
2. Job stress	0.152^***^	1			
3. Job demands	0.118^***^	0.540^***^	1		
4. Control over work	−0.70^***^	−0.587^***^	0.225^***^	1	
5. Sleep quality	−0.103^***^	−0.137^***^	−0.112^***^	0.078^***^	1

*** p < 0.001.

We also carried out a hierarchical linear regression analysis to assess the effects of sex, age, educational attainment, marriage status, household registration, years of service, employment status, sector of activity, financial difficulties, job stress, and sleep quality on HRPL in the sample, and in our models, job stress and sleep quality are used as predictive variables, while other variables are control variables. As the data pattern for HRPL was skewed, the variable was log-transformed and then included in the regression calculation. The variables age, sex, educational attainment, marriage status, household registration, years of service, employment status, sector of activity, and financial difficulties were included in Model I. Job stress was included in Model II, and sleep quality was included in Model III. In the analysis, Model I could explain 1.5% of the variance in HRPL, whereas Model II explained 2.8%. When the job stress variable was added, the model III explained 3.6% of the variance in HRPL. Furthermore, as shown in Table 4, predictors of HRPL in Model III were age, marriage status, household registration, financial difficulties, job stress, and sleep quality. The inclusion of sleep quality in Model III resulted in a significant decrease in the β for occupational stress (0.164–0.150), which suggests that sleep may play a mediating role between job stress and HRPL. Table 4 presents the results of hierarchical multiple regression analysis in further detail.

**Table 4:** Hierarchical multiple regression analysis predicting health-related productivity loss among workers in R&D companies.

	**Model I**		**Model II**		**Model III**	
	β	***P***	β	***P***	β	***P***
Age	−0.006	**0.014**	−0.005	**0.032**	−0.005	**0.030**
Sex	0.000	0.986	0.007	0.785	0.000	0.991
Educational attainment	−0.003	0.804	−0.001	0.902	0.003	0.815
Marital status	0.082	**0.009**	0.084	**0.007**	0.086	**0.006**
Household registration	−0.052	**0.004**	−0.052	**0.004**	−0.058	**0.001**
Years of service	−0.007	0.739	−0.012	0.540	−0.016	0.413
Employment status	0.000	0.988	−0.001	0.947	−0.002	0.936
Sector of activity	−0.002	0.903	−0.004	0.773	−0.006	0.638
Financial difficulties	−0.071	**<0.001**	−0.056	**0.001**	−0.044	**0.010**
Job stress			0.164	**<0.001**	0.150	**<0.001**
Sleep quality					−0.019	**<0.001**
R^2^	0.015		0.028	0.036		
Adjusted R^2^	0.012		0.025	0.033		
ΔR^2^	0.015		0.013	0.008		
ΔF	5.330^***^		43.605^***^	26.092^***^		

*** *p* < 0.01. Bold values mean *p* < 0.05.

**Figure 2:**
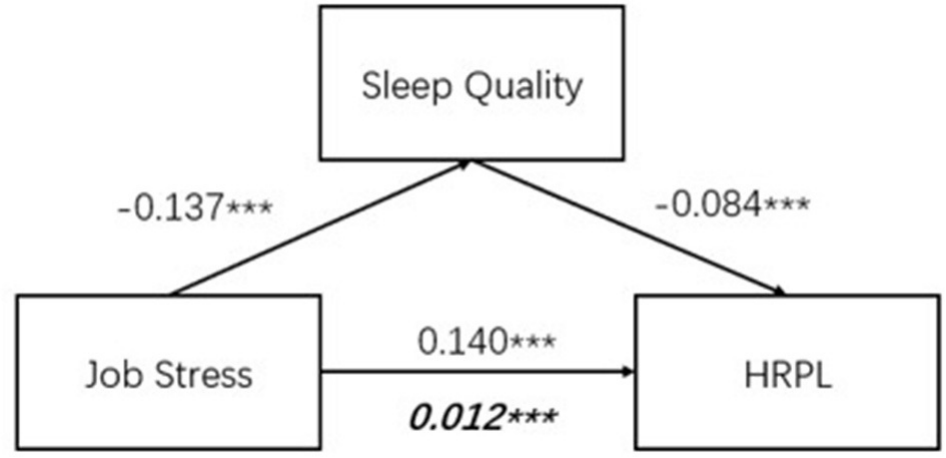
Mediating effect of sleep quality in job stress and health productivity loss. Partial mediating effect 7.57%. ***p < 0.01.

Finally, referring to Model 4 prepared by Hayes, mediation analysis was performed to assess the mediating effect of sleep quality on the association between job stress, as the independent variable, and HRPL, as the dependent variable. We used a simple mediation model, with one independent variable (job stress), one mediator (sleep quality), and one outcome variable (HRPL), to investigate mediation using estimated regression equations. The choice of sleep quality as a mediator emerged from conceptual theory, which was based on prior research on the relationship of sleep quality as a potential mediator of HRPL. The indirect effect was calculated by multiplying the path from the independent variable to the mediator variable by the path from the mediator variable to the dependent variable. Figure 2 shows that the standardized regression coefficient between job stress and HRPL was statistically significant, as was the standardized regression coefficient between sleep quality and HRPL. The standardized indirect effect (−0.137) × (−0.084) = −0.012 was significant (0 is not within the range of the 95% confidence interval). Thus, the indirect effect was statistically significant.

## 4 Discussion

The current study sought to analyze the mediating effect of sleep quality on the relationship between job stress and HRPL. The results showed that job stress can have an effect on HRPL through sleep quality. In other words, the extent to which occupational tension affects HRPL decreases when sleep quality is taken into account, but its effect can be indirectly observed in that improving sleep quality can reduce the extent of HRPL. Furthermore, our results showed that sleep quality is a significant negative predictor of HRPL, which is in line with the results of previous studies (4). Furthermore, job stress was a significant negative predictor of sleep quality, which contributes to the growing evidence that occupational stress is associated with sleep quality outcomes (e.g., increased anxiety and depression) (24–28).

From a practical point of view, the results suggest that organizations seeking to improve HRPL for their workers should aim to alleviate occupational tensions among their employees. Given the multi-causal nature of occupational tension, this means giving workers sufficient autonomy and control over their work and improving working conditions (29–31). Furthermore, in interventions aimed at enhancing employee HRP, while addressing occupational stress, it is advisable to incorporate programs that focus on improving sleep quality. This ensures that the potential positive impact of reducing job stress on health productivity can be facilitated through the avenue of enhanced sleep quality.

Regarding the comparison of HRPL according to different individual characteristics, younger people with a shorter length of service showed higher HRPL levels, which is due to higher levels of job control with longer length of service and greater skills, which delays the development of HRPL to some extent. There was no significant difference in the comparison of HRPL between different types of work, although production operators had a higher prevalence of both job stress and sleep disturbance compared work mental work-oriented management and R&D workers, suggesting that greater attention should be paid to the production conditions of production operators (32). Financial difficulties showed a significant effect on occupational stress, sleep quality, and HRPL. Greater financial difficulties were related to a greater likelihood of job stress, poor sleep quality, and higher levels of HRPL, and this finding was consistent with previous research (33–35). HRPL may also lead to the occurrence of increased medical expenses and reduced labor income, which may result in a vicious circle of further financial difficulties, requiring sufficient attention from organizations.

The results of this study should be considered within the context of certain limitations. First, the cross-sectional approach to the study hinders a broad understanding of the dynamics of variables over time. Second, convenience sampling makes it unclear to what extent the sample in this study is representative of the overall picture. Third, the online survey format may have caused some selection bias such that the sample tended to include workers familiar with online questionnaires. Finally, focusing on the target comes at the expense of weakening extrapolation; the survey of R&D companies in the Minhang District of Shanghai limits our understanding regarding the situation of workers in other parts of China.

To address the limitations of this study, the researchers suggest that future studies focus on selecting samples for comparison in regions with different levels of development, as well as a more detailed disaggregated study of workers in R&D-type positions and those in operational positions. We also recommend that longitudinal surveys be conducted with a view to uncovering situations where the dynamics of variables change in relation to each other.

## 5 Conclusions

Our findings are consistent with those of previous studies (2, 36) and have important practical implications, suggesting that organizations should pay greater attention to certain groups of workers who experience sleep disturbances in the presence of job stress, thereby preventing HRPL. The findings of the study are useful for professionals and managers working in the field of occupational health to identify factors that influence job stress, sleep disturbance, and HRPL among workers. To provide better occupational health services, occupational health professionals and human resource management teams must provide appropriate occupational conditions, paying particular attention to the variables in this study to positively influence the wellbeing of workers while improving productivity.

## Data availability statement

The raw data supporting the conclusions of this article will be made available by the authors, without undue reservation.

## Author contributions

YS: Data curation, Investigation, Writing – original draft. MW: Investigation, Writing – review & editing. QZ: Data curation, Writing – review & editing. JY: Investigation, Writing – review & editing. JG: Supervision, Writing – review & editing. JD: Writing – review & editing.
